# Fusion of clinical magnet resonance images and electronic health records promotes multimodal predictions of postoperative delirium

**DOI:** 10.1038/s41598-025-31693-9

**Published:** 2025-12-26

**Authors:** Niklas Giesa, Andrea Dell’Orco, Michael Scheel, Carsten Finke, Felix Balzer, Claudia Doris Spies, Maria Sekutowicz

**Affiliations:** 1 Institute of Medical Informatics, Charité - Universitätsmedizin Berlin, 10117 Berlin, Germany; 2 Institute of Neuroradiology, Charité - Universitätsmedizin Berlin, 10117 Berlin, Germany; 3 Experimental Neurology, Charité - Universitätsmedizin Berlin, 10117 Berlin, Germany; 4 Department of Anesthesiology and Intensive Care Medicine (CCM, CVK), Charité - Universitätsmedizin Berlin, 13353 Berlin, Germany; 5 Berlin Institute of Health at Charité – Universitätsmedizin Berlin, BIH Biomedical Innovation Academy, Charitéplatz 1, 10117 Berlin, Germany

**Keywords:** Machine learning, Postoperative delirium, Feature fusion strategies, Electronic health records, Brain morphometry, Neuroscience, Translational research

## Abstract

Brain morphometry derived from clinical imaging has an underexplored potential for the multimodal prediction of postoperative delirium (POD), an acute encephalopathy that can lead to long-term adverse outcomes or death. This study conducted a comprehensive analysis of patient trajectories, integrating magnetic resonance imaging (MRI) data and electronic health records (EHRs) across two general surgical cohorts. We applied univariate test methods and linear mixed-effects models correcting for confounding. Non-linear multi-layer perceptrons (MLPs), boosted decision trees, and logistic regressions were trained on EHR data, brain morphometry measures, and their multimodal fusion to predict POD. Age-adjusted correlations identified cortical thickness of temporal gyri, as well as thalamic and brainstem volumes to be POD-relevant neuroanatomical features. MLP models demonstrated robust predictive capability, achieving notably high performances up to 86% AUROC (area under the receiver operating characteristic). Multimodal fusion yielded pronounced benefits in less critically ill patients. MLP model weights showed high predictive potential for cerebral atrophy in higher-order cortical regions, including the temporal pole, superior frontal gyrus, and the insula. These findings reveal the previously unrecognized potential of clinically derived brain morphometry in enhancing early multimodal predictions of POD. A better understanding of brain vulnerability in POD may translate into improved clinical decision making based on multimodal health care data.

**Supplementary Information:**

The online version contains supplementary material available at 10.1038/s41598-025-31693-9.

## Introduction

Delirium is a distressing neuropsychiatric syndrome characterized by acute disturbances in consciousness, cognition, and attention^1^. Postoperative delirium (POD), occurring after major surgical procedures, is associated with adverse outcomes, such as prolonged hospitalization or death. Prevalence rates span from 5 to 52%^2^. The etiology of POD is multifactorial, with both predisposing and precipitating factors contributing to its acute onset^2,3^. Predisposing factors, such as preexisting cognitive impairment or advanced age, confer baseline vulnerability, while precipitating factors relate to perioperative conditions, including the surgical procedure^2^.

POD manifests through heterogenous levels of vigilance, neuropsychological, and psychotic symptoms which fluctuate in presence and severity demanding close monitoring and early assessment^2,4^. Previous studies indicate that structural brain changes may increase vulnerability to POD^5^. Patients with cerebral atrophy are predisposed to suffer from longterm cognitive decline^6^. Vulnerability to delirium may be facilitated by preexisting neuroanatomical changes resulting in neuronal dysfunction and network disintegration^7^. Such pre-morbidity in POD patients has been identified as decreased white matter integrity and increased gray matter atrophy^8–10^. Previous studies have been restricted to specific patient cohorts at risk, such as elderly patients undergoing major surgical procedures^8^. Thus, the associated structural brain changes may be age-specific or restricted to patients with preexisting cognitive impairment^9^.

While previous studies have developed non-linear machine learning (ML) prediction models^4,11,12^ that outperform standard statistical methods, these models rarely translate into clinical practice. Advanced ML approaches that integrate routinely collected clinical data from multiple modalities may overcome this limitation, as Mohsen et al.^13^ illustrate various fusion strategies. Such data fusions are applied either early in the feature space or later when outputting prediction probabilities. To the best of our knowledge, we are the first to utilize neuroanatomical features extracted from preoperative clinical MRIs to use premorbid structural brain changes for POD prediction. We systematically explore the predictive value of combining these MRI features with EHR data in two distinct general surgical cohorts. Since POD may often be undiagnosed^14^, we defined an endpoint based on agitation and pharmacological treatment for delirium for intensive care patients. Hereby, we complement standard delirium assessment tools for POD labeling which are routinely used postoperatively. Interpretation of multimodal ML techniques is augmented by linear mixed-effect models (MEM) correcting for covariates allowing further insights into the pathomechanisms of POD.

## Methods

### Study population, endpoint definitions, and data extractions

**Fig. 1:**
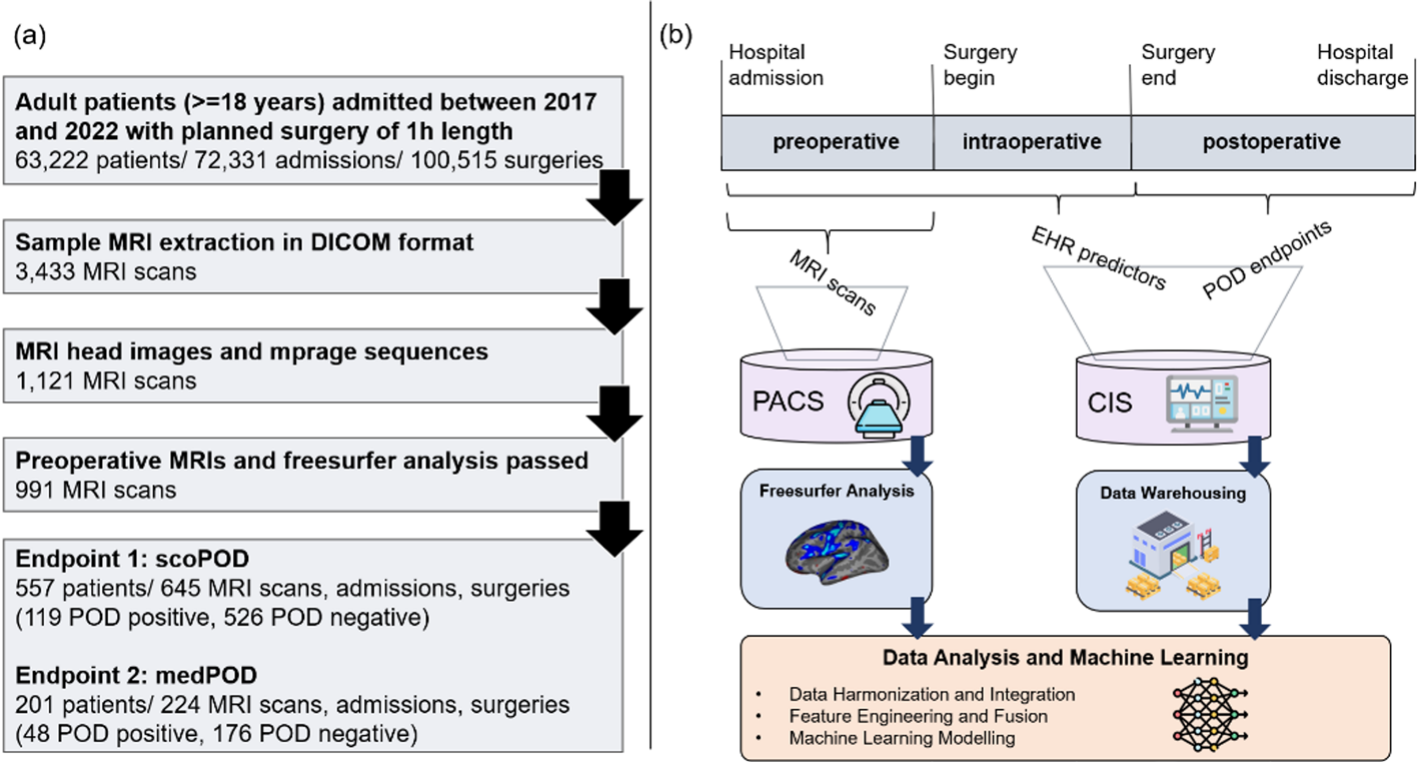
Inclusion criteria for endpoint definitions **(a)**, methodology and data flow **(b).** We included data of all adult patients between 2017 and 2022 with estimated surgery length of at least one hour. MRI headers from DICOM metadata were filtered for head-scans, MPRAGE sequences, and scanning time. Preoperative MPRAGE sequences that did not pass automated segmentation (e.g. extreme movement) were excluded (shown in **a**). We divided the hospital stay into pre-, intra-, and postoperative time phases. MRI scans were extracted from the picture archiving and communication system (PACS), EHR data were extracted from the clinical information system (CIS) for integrated data analysis and the application of machine learning (shown in **b**).

We included all patients (aged > = 18) who underwent surgery between 2017 and 2022 if the estimated surgery duration was > = 1 h, initially resulting in EHR-data from 63,222 patients (see Fig. 1a). All data for this single-center study were provided by three different sides at Charité, a large German university hospital. This study is the first in our medical institution to leverage routinely acquired MRIs. As no standard data pipeline was available, we extracted a random sample of preoperative MRIs from the picture archiving and communication system (PACS) without additional capabilities (e.g., no information on POD assessments or type of scans) (see Fig. 1b**)**. This procedure resulted in 3,344 heterogenous de-identified MRI scans.

Preoperative MRI was obtained for broad clinical indications and could be unrelated to subsequent surgical procedures (e.g., screening for intracranial metastases, suspected stroke, oncologic staging, surveillance/follow-up, or headache/seizure workup, as well as performed for neurosurgical planning). Subsequently, MRI headers were filtered for cranial scans, acquisition times, and existences of T1-weighted MPRAGE sequences yielding a cohort of 991 MRI scans (see Fig. 1a).

The clinical information system (CIS) stored pre- and intraoperative EHRs that were de-identified and archived in a Data Warehouse (DWH)^15^ allowing data harmonization (see Fig. 1b). We used these EHRs to define predictors, endpoints, and to link clinical predictors to MRIs from the PACS. POD was defined as a binary endpoint variable (1 = delirious, 0 = non-delirious) according to two definitions.

All surgical patients are routinely screened for delirium with the Nursing Delirium Screening Scale (Nu-DESC) preoperatively before the anesthesia and postoperatively during post-anesthesia care inside the recovery room. Patients transferred directly to the ICU are assessed with the Confusion Assessment Method for the Intensive Care Unit (CAM-ICU) at admission and at least three times per day, according to institutional standards. For the score-based POD definition (scoPOD), CAM-ICU and Nu-DESC^16^ assessments were used to classify patients as delirious (at least one Nu-DESC > 0 or positive CAM-ICU) or non-delirious (all Nu-DESC = 0 or all CAM-ICU negative). 645 scans for 557 patients are covered by scoPOD.

The second medication-based endpoint definition (medPOD) was based on agitation and pharmacological treatment for delirium. ICU patients are routinely assessed for the level of agitation along the standard Richmond Agitation Sedation Scale (RASS)^17^ for critically ill patients. Those who postoperatively scored a RASS of > 1 and subsequently received any of the medications Haloperidol, Clonidine, Dexmedetomidine, Pipamperone, or Risperidone were labeled as delirious. RASS assessment and given medication were required to be temporarily aligned to the same day (within a 24 h interval). Controls maintained a RASS of 0 and did not receive any aforementioned medications until discharge. Any other cases were excluded from the medPOD definition, resulting into 224 scans for 201 patients.

### Data preprocessing

As routinely collected EHRs potentially follow skewed distributions^18^, we aggregated parameters with robust summary statistics as mean, median, 10th – and 90th percentile separately for the pre- and intraoperative phase. Our initial feature space covered parameters that were available for at least 10% of patients. Laboratory values (lymphocytes, CRP, etc.), medications (propofol, norepinephrine, etc.), vital signs (heart rate, spo2, etc.), and device settings (FiO2, PEEP) resulted in 133 features (see **Extended Table** A1/A2 in Supplement B). To avoid estimation bias, the feature space excluded any data contributing to our two endpoint definitions medPOD or scoPOD, including medications commonly used to control for delirious symptoms. All data were normalized via z-transformation with statistics from the training set. ICD-based delirium labels would lack temporal information and might reflect documentation gaps rather than negative cases. Consequently, presence of diagnoses in the form of an ICD code^19^ were used to characterize our cohorts, but omitted for training ML models or endpoint definitions due to uncertain documentation times^20^. 

### Statistical analysis

We analyzed features towards binary endpoints with Mann-Whitney U (MWU) test statistics^21,22^. We report the AUC-0.5 with 0 as a chance-level, values near 1 as a strong positive, and values near − 1 as a strong negative effect. The Spearman correlation coefficient (ρ) was used to describe the association of clinical parameters with age, we defined the effect strength similarly with levels of 0, near 1 or −1^23^.

To account for age as a confounder in MRI-based brain morphometry, we configured multivariate linear mixed-effect models (MEMs)^24^. MEMs had varying intercepts for single feature effects as $\:C\left(POD\right)\:\sim\:age+feature+1|patient$. Here, the POD endpoint functions as the dependent variable, age as fixed effects in conjunction with the feature of interest. To correct for multiple surgeries and multiple MRI scans, patient identifiers were integrated as random effects. We report age-adjusted p-values and a corresponding coefficient β(f) indicating effect directions.

Statistical significance was assessed using false discover rate (FDR) corrections (alpha = 0.05)^25^. For reporting the effects of binary variables with POD, we used the odds ratio (OR) on a logarithmic scale as ln(OR) with large deviations from 1 indicating strong associations.

### MRI analysis

All DICOMs were converted to NIfTIs using dcm2niix and segmented by the FreeSurfer v7.4.1 recon-all pipeline^26^ Morphometry measures were computed using Desikan-Killiany atlas-based parcellation^27^. We visually assessed all results to identify missegmentation, administration of contrast agents or anatomical aberrations, such as general atrophy or tissue lesions. In the case of brain abnormality, only healthy contralateral hemispheres were selected for analyses. Otherwise, one hemisphere was randomly selected resulting in 358 right – and 287 left (scoPOD) as well as 91 right – and 133 left (medPOD) hemispheres. To account for the effect of intracranial volume, volume estimates were normalized by division with the estimated total intracranial volume. The final MRI-related feature set was composed of 184 volumes, 70 thickness features, and 72 area features.

### Machine learning and fusion strategies

We trained three ML techniques comprising logistic regression (LR), gradient boosted trees (BT), and multi-layer perceptron (MLP) architectures. While LR assumes linear relationship, BT and MLP represent non-linear problems^28,29^ with MLPs functioning as universal approximators^30^ stacking perceptrons (nodes) on interconnected layers.

To handle different data modalities, we deployed two fusion strategies^13^. In “early fusion”, we enhanced our input feature space by ingesting selected measures from both types. For “late fusion”, we trained separate models for each modality (MRI or EHR), combining the prediction outputs (see **Extended Figure** B1 in Supplement A). For BT and LR, model outputs (probabilities between 0 and 1) were simply mean-averaged. In the late fusion MLP, a linear layer integrates predictions from both models into a single output while learning the weights for both networks via backpropagation, also known as “joint fusion”. We additionally trained completely separate models for MRI and EHR features.

### Model configuration, training, and validation

For optimal model configurations (hyperparameters), a 3 × 3 nested cross-validation (CV)^31^ approach was implemented. Different sets of parameters are exhaustively validated via a Grid-Search^32^ on the inner-nested CV process and then applied to the outer-nested one. 1000x bootstrapping enabled estimations of 95% confidence interval (CI)^33^ for validation results.

The area under (AU-) the receiving operating characteristics (-ROC), and the precision recall curve (-PRC) evaluated performances^22^. As a cost function, we configured a weighted binary cross-entropy (BCE) loss to address class imbalance^34^. The final parameter space included regularization techniques, like BT pruning or L_1_-norm penalty for MLP and LR, in addition to general configurations (see **Extended Table** B2 in Supplement A). We trained models with subsets of features for different adjusted p-values thresholds (see **Extended Table** A3 in Supplement B).

## Results

### Cohort characteristics

**Table 1:** Descriptive cohort characteristics for two POD endpoints. Descriptive statistics are displayed as mean ± sd for numerical variables. For binary variables, the fraction of positive samples from all (n) are cited followed by the odds as (pos/neg samples). Adjusted p-values are derived from linear mixed-effect models (MEMs) incorporating age and the variable of interest as fixed effects, patient groups as random effects and POD as the independent variable. We highlight significant results with asterisks according to a FDR corrected alpha level. RASS: Richmond agitation sedation Scale, SOFA: sequential organ failure Assessment, SIRS: systemic inflammatory response Syndrome, urgency class N: ranges from *N* = 1 (immediate surgery required) to *N* = 5 (elective, planned procedure), anesthesia type of surgery: minor surgical procedures requiring sedation or anesthesia stand by.

	Endpoint 1: scoPOD				Endpoint 2: medPOD			
	All (*n* = 645)	POD Positive (*n* = 119)	POD Negative (*n* = 526)	*P*-Value	All (*n* = 224)	POD Positive (*n* = 48)	POD Negative (*n* = 176)	*P*-Value
General Information								
Age (years)	59.32 ± 16.23	65.01 ± 14.04	58.02 ± 16.43	< 0.05*	58.03 ± 15.61	62.08 ± 13.49	56.85 ± 16.02	< 0.05*
Sex (male/female)	0.78 (510/135)	0.71 (84/35)	0.81 (426/100)		0.69 (155/69)	0.59 (28/20)	0.72 (127/49)	
Number of Surgeries	2.27 ± 3.32	3.17 ± 4.04	2.06 ± 3.10		1.94 ± 2.23	3.19 ± 3.94	1.58 ± 1.18	
Length of Hospital Stay (days)	31.45 ± 3.10	44.7 ± 4.57	28.43 ± 2.22		34.25 ± 4.80	64.65 ± 6.90	25.4 ± 3.51	
Length of Anesthesia (hours)	3.10 ± 3.07	3.18 ± 3.79	2.77 ± 4.67		5.14 ± 2.97	5.72 ± 3.82	4.98 ± 2.29	
Length of Surgery (hours)	1.52 ± 1.16	1.52 ± 1.16	1.48 ± 1.19		1.98 ± 1.34	2.05 ± 1.32	1.7 ± 1.38	
Length of Recovery Room Stay (hours)	4.82 ± 2.66	5.18 ± 2.42	2.81 ± 3.28	< 0.001***	5.14 ± 2.78	5.28 ± 2.71	4.58 ± 3.03	
Clinical Assessment								
Urgency Class N	4.24 ± 1.79	4.12 ± 1.50	4.15 ± 1.47		4.12 ± 1.44	3.41 ± 1.81	4.32 ± 1.24	
ASA Status	1.93 ± 1.22	1.97 ± 1.40	1.92 ± 1.40	< 0.001***	2.06 ± 1.34	2.12 ± 1.21	1.82 ± 1.69	< 0.001***
SOFA	4.25 ± 3.18	5.48 ± 3.24	3.34 ± 2.83	< 0.05*	4.95 ± 3.22	6.25 ± 3.05	3.87 ± 2.98	< 0.05*
RASS	−0.66 ± 1.00	−1.03 ± 1.19	−0.47 ± 0.83		−0.90 ± 1.14	1.08 ± 1.16	0.81 ± 1.12	< 0.001***
Minutes to 1 st POD assessment	25.53 ± 27.15	26.83 ± 26.49	25.47 ± 26.95		55.37 ± 63.15	54.21 ± 68.04	55.74 ± 67.44	
**Type of Surgery**								
Neurosurgical	0.56 (363/282)	0.49 (58/61)	0.58 (305/221)		0.60 (134/90)	0.27 (13/35)	0.69 (121/55)	< 0.001***
Visceral	0.06 (40/605)	0.07 (8/111)	0.06 (32/494)		0.05 (11/213)	0.08 (4/44)	0.04 (7/169)	
Anesthesia	0.07 (46/599)	0.12 (14/105)	0.06 (32/494)		0.06 (13/211)	0.08 (4/44)	0.05 (9/167)	
Cardiac	0.01 (9/636)	0.05 (7/113)	0.01 (3/523)		0.04 (8/216)	0.04 (6/42)	0.01 (2/175)	
**Predisposing Risk**								
Cardiovascular	0.24 (158/487)	0.31 (37/82)	0.23 (121/405)		0.57 (128/96)	0.30 (65/159)	0.28 (49/127)	
Non-Smoking	0.29 (193/452)	0.29 (35/84)	0.30 (158/368)		0.40 (89/135)	0.27 (13/35)	0.42 (74/102)	
Other Comorbidities	0.02 (10/635)	0.02 (4/117)	0.01 (6/520)		0.02 (4/220)	0.02 (1/47)	0.02 (3/173)	
Drinking	0.01 (5/640)	0.02 (3/116)	0.01 (2/521)		0.01 (2/222)	0.04 (2/46)	0.00 (0/176)	
**MRI Screening**								
Tumor	0.18 (114/531)	0.17 (20/99)	0.18 (94/432)		0.20 (45/179)	0.14 (6/42)	0.22 (39/137)	
Atrophy	0.07 (42/603)	0.09 (10/109)	0.06 (32/494)		0.08 (18/206)	0.08 (3/45)	0.08 (14/162)	
Lesion	0.43 (279/366)	0.34 (40/79)	0.45 (239/287)		0.53 (119/105)	0.52 (24/24)	0.54 (95/81)	
Contrast Agent	0.79 (512/133)	0.60 (70/49)	0.84 (442/84)		0.82 (184/40)	0.8 (38/10)	0.83 (146/30)	
**ICD Diagnose**								
Any Malignancy	0.50 (321/324)	0.49 (58/61)	0.50 (263/263)		0.51 (105/109)	0.45 (22/26)	0.53 (93/83)	
Renal Disease	0.37 (241/524)	0.61 (73/46)	0.32 (168/358)	< 0.001***	0.43 (97/127)	0.71 (34/14)	0.36 (63/113)	< 0.001***
Metazoic Tumor	0.28 (179/466)	0.31 (37/82)	0.27 (142/384)		0.29 (66/158)	0.22 (11/37)	0.31 (55/121)	
Delirium	0.19 (118/517)	0.85 (101/18)	0.03 (17/509)	< 0.001***	0.30 (68/156)	0.88 (42/48)	0.15 (26/150)	< 0.001***
Hemiplegia Paraplegia	0.20 (129/516)	0.23 (27/92)	0.20 (105/421)		0.28 (62/162)	0.29 (14/34)	0.27 (48/128)	
SIRS	0.14 (92/543)	0.52 (62/57)	0.06 (30/494)	< 0.001***	0.21 (46/178)	0.67 (32/16)	0.08 (14/162)	< 0.001***
Sepsis	0.12 (79/556)	0.16 (19/100)	0.11 (60/466)	< 0.05*	0.17 (38/186)	0.58 (28/20)	0.06 (10/166)	< 0.001***
Dementia	0.02 (13/632)	0.08 (9/110)	0.01 (4/517)	< 0.05*	0.04 (8/216)	0.04 (2/46)	0.03 (6/170)	

Cohort characteristics for the score-based endpoint scoPOD and the medication-based endpoint medPOD revealed POD prevalence rates of 18.44% and 21.43%, respectively **(**Table 1). Patients who met inclusion criteria for scoPOD underwent a mean of 1.23 delirium assessments with Nu-DESC or CAM-ICU scales. P-values refer to POD cases vs. controls per endpoint. POD patients were older (65.01 ± 14.04 years as mean ± sd for scoPOD, 62.08 ± 13.49 years for medPOD, *p* < 0.05) than controls (58.02 ± 16.43 years for scoPOD, 56.85 ± 16.02 years for medPOD, *p* < 0.05). Highly significant POD differences were observed in recovery room stay durations for scoPOD (5.18 ± 2.42 h for POD, 2.81 ± 3.28 h for controls, *p* < 0.001). Patients’ physical status and degree of agitation was significantly reduced for delirious medPOD patients (ASA 2.12 ± 1.21 POD, 1.82 ± 1.69 controls, *p* < 0.001; RASS 1.08 ± 1.16 POD, −0.81 ± 1.12 controls, *p* < 0.001).

When comparing cohorts, medPOD patients had 0.31 h longer stays in the recovery room, a decreased physical status (Δ of mean ASA = 0.13), were more prone to sequential organ failure (Δ of mean SOFA = 0.70), and less agitated (Δ mean RASS = 0.24). In both cohorts, the most prominent surgical procedure was neurosurgery, reaching a significant difference for medPOD labels (ln(OR) = 1.71, *p* < 0.001). Visual MRI screening properties, such as general atrophy, did not show significant differences for POD (see Table 1). Confirming POD labeling, we observed highly significant correlation with ICD encoded delirium (ln(OR) = 2.10, *p* < 0.001 scoPOD, ln(OR) = 2.92, *p* < 0.001 medPOD). Additional characteristics are included in **Extended Table** B3 and **Extended Results** B1 in Supplement A).

### MRI and EHR single feature importance

**Fig. 2:**
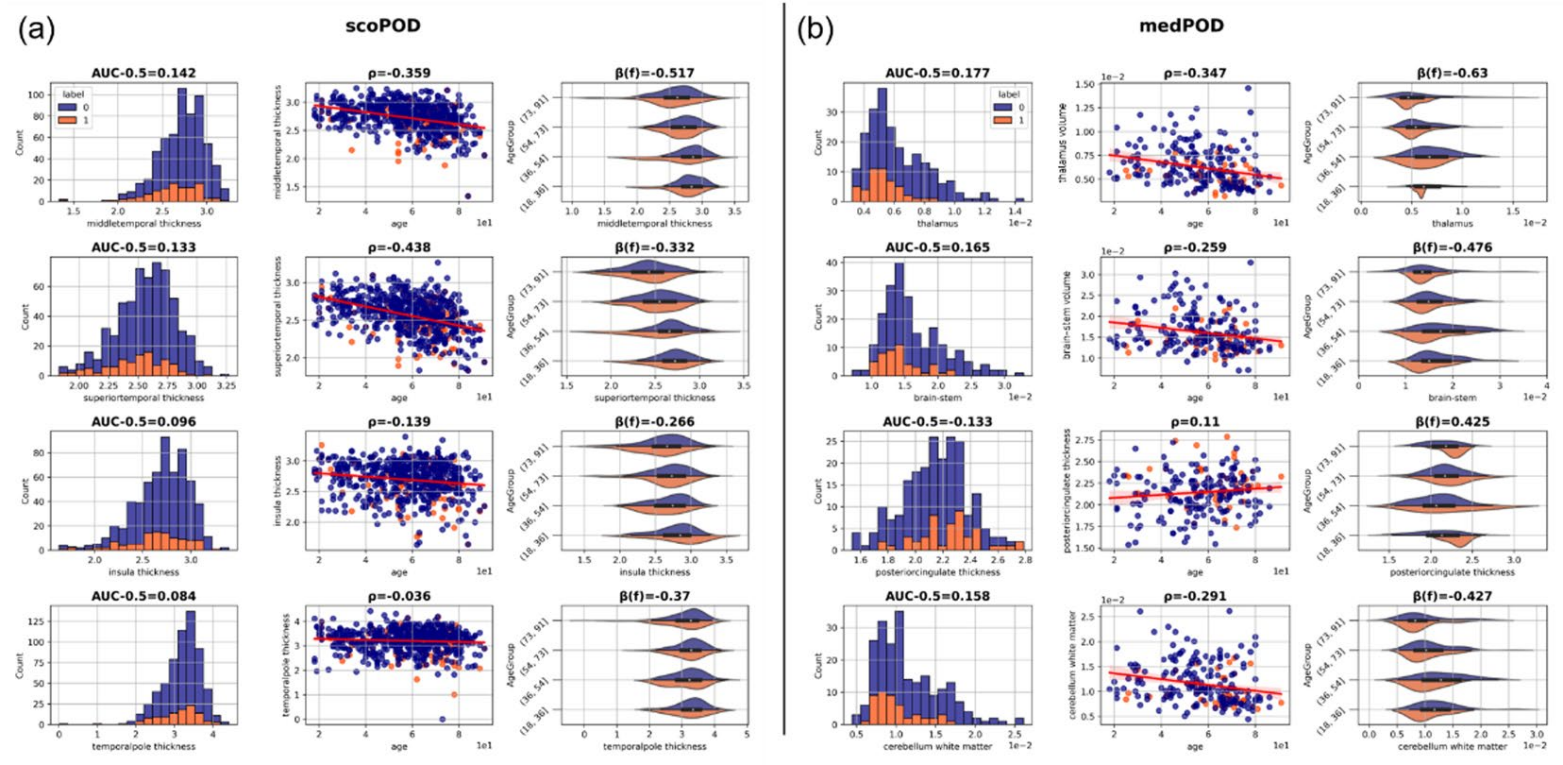
Top 4 single MRI feature importance for endpoint scoPOD **(a)** and medPOD **(b).** We display most significant results according to an age-adjusted p-value derived from mixed-linear-effects models (MLEMs). For each panel (**a**) or (**b**), the first column displays the distribution of feature values for POD cases (label = 1, orange) and controls (label = 0, blue) including AUC-0.5 as an effect size derived from Mann-Whitney U (MWU) statistics. The second column shows the correlation between the feature and age providing the Spearman Rank correlation coefficient ρ. The last column depicts age-group specific differences in distributions of POD cases and controls for each feature citing the corresponding coefficient β(f) retrieved from MEMs. The black boxes inside violin plots show a boxplot from the entire distribution per age group where the white dot indicates the median.

**Table 2:** Single feature importance for MRI and EHR features per endpoint. Univariate results from Mann-Whitney U (MWU) test statistic define discriminability of [POD] endpoints with unadjusted p-value and AUC-0.5 as effect size. Spearman ranks provide correlation coefficient (ρ) calculated between feature values and [Age] including p-value under the null-hypothesis of zero-coefficients. Linear mixed-effect models (MEM) were fitted with [POD] as dependent variable, feature values as fixed effects, patient-MRI hierarchy as random effects. MEMs provide age-adjusted p-values and a corresponding coefficient β(f). Data availability shows the fraction of available feature values from all, adjacent to the odds of (available/missing) values. EHRs must be aggregated with either the sum, median (md), mean (me) for preoperative (pre) or intraoperative (intra) time phases. FDR corrected significant results are highlighted in bold and italics.

		Mann-Whitney U		Spearman Correlations		Linear Mixed Effects		
		*P*-Value [POD]	AUC-0.5 [POD]	*P*-Value [Age]	Coefficient ρ [Age]	Adj. *P*-Value [POD]	Coeff. β(f) [POD]	Data Availability % (avail/miss)
Endpoint 1 scoPOD (*n* = 645)								
**MRI Features**	Middle Temporal Thickness	**7.26E-06**	−0.142	***2.14E-19***	−0.359	***3.25E-05***	−0.517	1.00 (645/0)
	Superior Temporal Thickness	**2.60E-05**	−0.133	***5.57E-29***	−0.438	***7.12E-04***	−0.332	1.00 (645/0)
	Insula Thickness	2.37E-03	−0.096	***6.99E-04***	−0.139	6.22E-03	−0.266	1.00 (645/0)
	Temporal Pole Thickness	8.32E-03	−0.084	3.87E-01	−0.036	7.91E-03	−0.370	1.00 (645/0)
	Superior Frontal Thickness	4.78E-04	−0.111	***5.25E-14***	−0.303	9.26E-03	−0.273	1.00 (645/0)
	Pericalcarine Thickness	5.22E-01	−0.020	***3.32E-08***	−0.225	1.24E-02	+ 0.580	1.00 (645/0)
	Entorhinal Thickness	1.39E-02	−0.078	***4.03E-05***	−0.168	1.74E-02	−0.343	1.00 (645/0)
	Parahippocampal Thickness	6.33E-02	−0.059	6.55E-03	−0.112	1.89E-02	−0.325	1.00 (645/0)
	White Matter Hyperintensities	***8.22E-05***	−0.125	***5.47E-28***	+ 0.430	2.22E-02	0.355	1.00 (645/0)
	Pars Opercularis Area	7.98E-02	−0.056	5.47E-01	−0.025	2.30E-02	−0.362	1.00 (645/0)
**EHR Features**	Sodium in Blood [mmol/l] (pre, me)	1.18E-03	+ 0.106	6.83E-02	−0.076	***1.14E-06***	+ 0.018	0.86 (557/88)
	Erythrocytes in Blood [pl] (pre, md)	***1.00E-05***	−0.144	2.35E-02	−0.095	***1.21E-06***	−0.093	0.87 (561/84)
	CRP in Blood [mg/l] (pre, me)	3.19E-05	+ 0.143	1.09E-01	0.073	***1.24E-06***	+ 0.001	0.72 (466/179)
	Hemoglobin [g/dl] (pre, md)	***1.19E-05***	−0.143	1.58E-01	−0.059	***1.99E-06***	−0.030	0.87 (564/81)
	Hematocrit [%] (pre, md)	2.79E-05	−0.137	2.65E-01	−0.047	***3.22E-06***	−1.092	0.87 (561/84)
	Heart Rate [1/min] (pre, 90th -p)	2.13E-04	+ 0.125	1.03E-03	−0.141	***4.07E-06***	+ 0.004	0.81 (521/124)
	Pulse [1/min] (pre, 90th -p)	2.71E-04	+ 0.133	3.77E-03	−0.139	***6.36E-06***	+ 0.004	0.66 (424/221)
	Respiratory Rate [1/min] (pre, me)	5.58E-05	+ 0.164	7.97E-01	−0.014	2.69E-05	+ 0.010	0.53 (343/302)
	Norepinephrin [mg] (intra, sum)	3.12E-02	+ 0.078	8.58E-01	−0.009	3.07E-05	+ 0.324	0.67 (431/214)
	Fluids Given [ml] (intra, sum)	2.06E-04	−0.031	8.13E-01	0.011	8.00E-05	−0.003	0.74 (480/165)
Endpoint 2 medPOD (*n* = 224)								
**MRI Features**	Thalamus Volume	***2.79E-04***	−0.177	***1.98E-07***	−0.347	***2.39E-04***	−0.630	1.00 (224/0)
	Brainstem Volume	***6.80E-04***	−0.165	***1.31E-04***	−0.259	3.50E-03	−0.476	1.00 (224/0)
	Posterior Cingulate Thickness	6.28E-03	−0.133	1.11E-01	0.110	4.38E-03	0.425	1.00 (224/0)
	Cerebellum White Matter	1.11E-03	−0.158	***1.63E-05***	−0.291	6.21E-03	−0.427	1.00 (224/0)
	Inferior Temporal Area	9.56E-02	−0.081	8.65E-02	−0.118	7.85E-03	+ 0.502	1.00 (224/0)
	Rostral Anterior Cingulate Volume	4.64E-03	−0.138	2.36E-02	+ 0.155	8.83E-03	+ 0.409	1.00 (224/0)
	Ventral Diencephalon	6.22E-03	−0.133	***2.12E-07***	−0.346	1.09E-02	−0.573	1.00 (224/0)
	Subcortical Gray Matter volume	7.39E-03	−0.130	***1.33E-07***	−0.352	1.54E-02	−0.450	1.00 (224/0)
	Fusiform Area	9.37E-02	−0.081	3.38E-01	−0.066	1.70E-02	+ 0.526	1.00 (224/0)
	Entorhinal Area	4.66E-03	−0.138	2.18E-01	0.085	2.08E-02	+ 0.541	1.00 (224/0)
**EHR Features**	Erythrocytes in Blood [pl] (pre, me)	***5.29E-08***	−0.266	9.74E-02	−0.115	***2.49E-09***	−0.185	0.92 (205/19)
	Hemoglobin [g/dl] (pre, md)	***1.17E-07***	−0.259	2.86E-01	−0.074	***7.00E-09***	−0.060	0.92 (205/19)
	Hematocrit [%] (pre, md)	***8.93E-07***	−0.240	4.13E-01	−0.057	***4.67E-08***	−2.057	0.92 (205/19)
	CRP in Blood [mg/l] (pre, me)	***1.75E-06***	+ 0.246	4.53E-02	+ 0.152	***1.08E-07***	+ 0.003	0.76 (170/54)
	Pulse [1/min] (pre, 90th -p)	1.82E-05	+ 0.220	6.34E-01	−0.035	***2.22E-06***	+ 0.007	0.83 (186/38)
	SaO2 [%] (pre, 10th -p)	***8.46E-06***	−0.227	3.42E-03	−0.211	***4.22E-06***	−0.047	0.83 (186/38)
	Norepinephrin [mg] (intra, sum)	1.23E-03	+ 0.170	2.55E-01	+ 0.082	2.20E-05	+ 0.355	0.85 (190/34)
	Fluid Given [ml] (pre, sum)	***1.76E-08***	+ 0.273	5.40E-01	+ 0.047	4.18E-05	+ 0.000	0.75 (167/57)
	ASA status (pre, me)	8.71E-05	+ 0.223	1.30E-04	+ 0.290	8.20E-05	+ 0.140	0.73 (164/60)
	Pulse [1/min] (intra, md)	4.74E-04	+ 0.174	8.18E-01	+ 0.016	3.35E-04	+ 0.006	0.91 (203/21)

MRI-derived morphometry features, such as the middle temporal- and superior temporal thickness were significantly correlated with scoPOD (MWU *p* = 7.26E-06, AUC-0.5= −0.142; *p* = 2.60E-05, AUC-0.5= −0.133) as well as with age (Spearman *p* = 2.14E-19, ρ= −0.359; *p* = 5.57E-29, ρ= −0.438) (see Table 2; Fig. 2a). Decreased cortical thickness resulted in increased probabilities of POD and occurred rather in elderly, than in younger patients.

Multivariate MEMs showed that cortical thickness of middle as well as the superior temporal cortex remained significantly associated with scoPOD when adjusting for age (adj. *p* = 3.25E-05, β(f)= −0.517; adj. *p* = 7.12E-04, β(f)= −0.332). Decreased cortical thickness in POD was preserved, when dividing patients into equally-sized age groups (negative MEM coefficient β(f), see Fig. 2a). Measures of white matter hypointensities expressed significant univariate effects on scoPOD (MWU *p* = 8.22E-05, AUC-0.5= −0.125) and age (Spearman *p* = 5.47E-28, ρ = 0.430).

MWU analysis of EHR features highlighted preoperative measures of anemia (hemoglobin: p-value = 1.19E-05; erythrocytes: *p* = 1.00E-05) and infection parameters (CRP: *p* = 3.19E-05). These were significant after age-corrections (MEM erythrocytes: adj. *p* = 1.21E-06; hemoglobin: adj. *p* = 7.00E-09, hematocrit adj. *p* = 3.22E-06; CRP: adjusted *p* = 1.24E-06).

For medPOD, subcortical MRI features, such as thalamus and brainstem volume, were significantly associated with POD (thalamus volume, *p* = 2.79E-04, AUC-0.5 = −0.177; MWU; brainstem volume *p* = 6.80E-04, AUC-0.5 = −0.165; MWU). After age-correction, the thalamus volume remained significantly associated with POD (adj. *p* = 2.39E-04) (see Table 2; Fig. 2b). Additionally, multivariate MEM analysis indicated significant associations of EHR features and blood parameters like low levels of erythrocytes (adjusted *p* = 2.49E-09, β(f) = −0.185), hemoglobin (adjusted *p* = 7.00E-09, β(f) = −0.060), hematocrit (adjusted *p* = 4.67E-08, β(f) = −2.057), and increased CRP (adjusted *p* = 1.08E-07, β(f) = 0.003) (see Table 2).

### Machine learning results

**Fig. 3:**
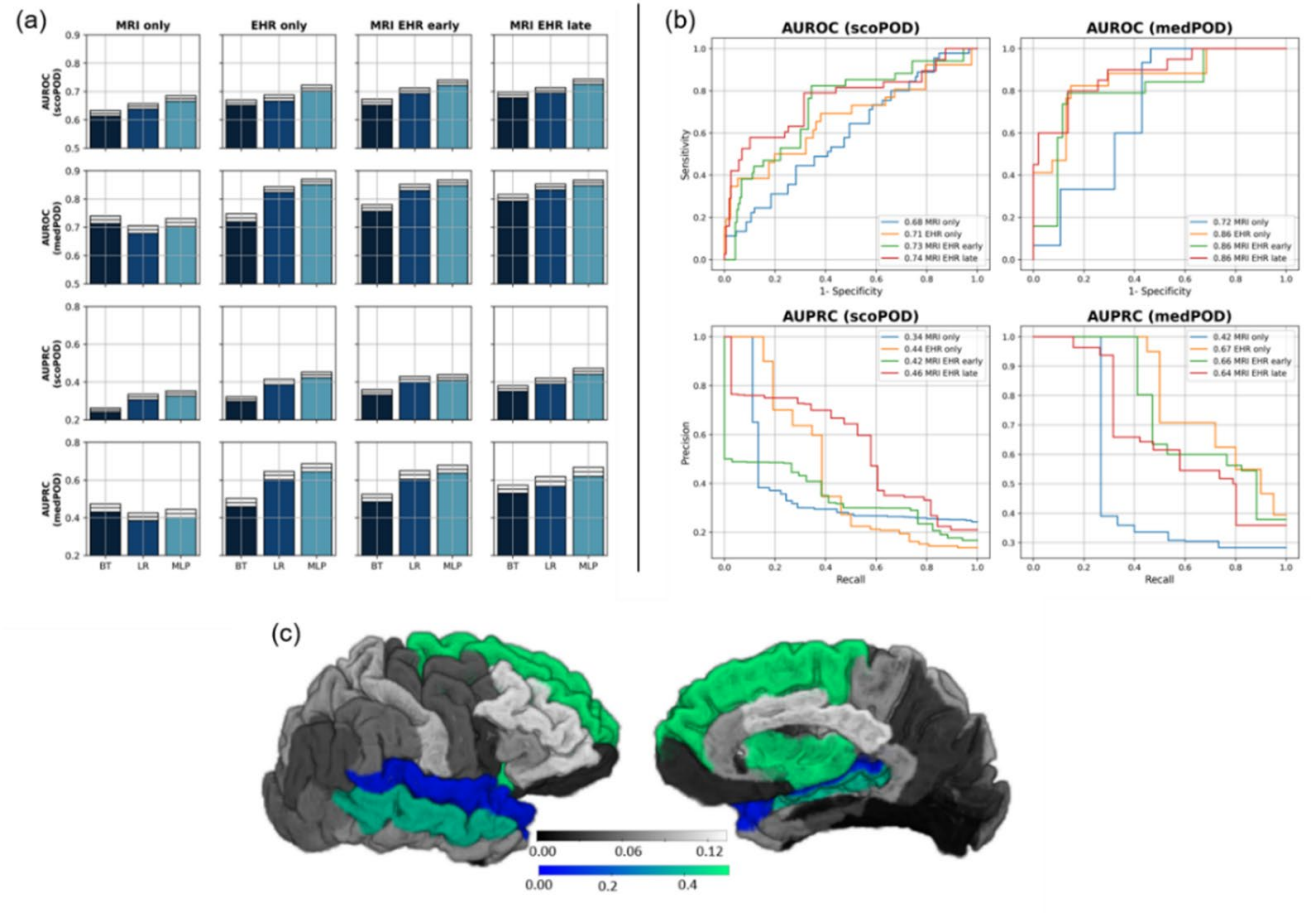
Performance metrics across machine learning models and fusion types **(a)** or for best performing model **(b)** as well as importance of MRI features for scoPOD **(c).** Area under (AU-) the receiver operating characteristics (-ROC) or the precision recall curve (-PRC) for trained and 3x cross-validated machine learning methods applied to predict endpoint scoPOD (*n* = 645) or medPOD (*n* = 224). Gradient boosted trees (BT), logistic regression (LR), and multi-layer perceptrons (MLPs) are included. Panel **a** shows metrics across models and fusion types either including magnetic resonance imaging features (MRIs), or electronic health records (EHRs) only, or combining these two modalities (early, late). 95% confidence intervals are drawsn as white boxes in (a) on top of bars calculated on 1000x bootstrapped validation folds. In panel b, trained MLPs are selected to draw AU curves showing mean performances across validation sets. In panel **c**, the absolute value of AUC-0.5 derived from MWU (grey scale) and absolute model weights (MW) from best MLP classifier (blue-green scale) trained with MRI only are shown. Anatomical segmentation across right hemisphere according to DKT-Atlas is displayed from temporal and medial. Overlay colored green-blue indicates model weights. Underlaying absolute AUC metrics for blended areas are: superior frontal thickness 0.111, insula thickness 0.096, middle temporal thickness 0.142, superior temporal thickness 0.133, temporal pole thickness 0.084.

We evaluated LR, BT, and MLP models with AUROC and AUPRC metrics for both endpoints. Highest performance for the score-based scoPOD cohort was achieved by a late fusion MLP (AUROC 0.735 [0.726, 0.744] as mean, [95% CI]; AUPRC 0.456 [0.411, 0.472]), outperforming LR (AUROC 0.705 [0.695, 0.715]; AUPRC 0.404 [0.389, 0.420]) and BT (AUROC 0.722 [0.712, 0.32]; AUPRC 0.450 [0.436, 0.468]) within the same fusion type (see Fig. 3a). MLPs were superior to LR and BT for all fusion types (see **Extended Table** B4 in Supplement A). We observed overlapping CIs of AUROC between late- and early fusion of MLPs for scoPOD ([0.726, 0.744] vs. [0.722, 0.740]), but distinct differences to MLPs trained with one modality only (EHR only [0.703, 0.721], MRI only [0.666, 0.685]).

The best model that predicted the medical-based endpoint medPOD used EHR features only (AUROC 0.861 [0.851, 0.871]; AUPRC 0.665 [0.644, 0.687]) (see Fig. 3a). Here, the confidence was decreased due to similar metrics ranges yielded by combined fusion MLPs like early - (AUROC [0.847, 0.860]; AUPRC [0.636, 0.679]), or late fusion (AUROC [0.847, 0.867]; AUPRC [0.619, 0.668]). MLPs showed overall elevated validation metrics in contrast to other ML methods.

In Fig. 3b, corresponding AUROC and AUPRC curves describe model behaviors under varying prediction thresholds. Curves confirm that late fusion was favorable for scoPOD with a sensitivity of 0.81 and a specificity of 0.63 at the threshold where their sums maximize. MLPs trained solely on EHRs exceeded these metrics with 0.81 sensitivity and 0.82 specificity predicting medPOD.

### Model interpretation

Model weights (MW) from our best MLPs per endpoint revealed ante-hoc feature importance. The best late fusion scoPOD based MLP focused on intraoperative tidal volume (abs MW = 0.288), preoperative albumin blood levels (abs MW = 0.283), and erythrocytes counts (abs MW = 0.2).

Highest model weights were found in the late fusion MLP using MRI features for scoPOD and were assigned to temporal pole thickness (MW = 0.565), superior frontal gyrus (MW = 0.523), and insula thickness (MW = 0.504). These features also showed univariate MWU feature importance with effect strengths of 0.084 (*p* = 8.32E-03), −0.111 (*p* = 4.78E-04), and − 0.096 (*p* = 2.37E-03) AUC-0.5 (see **Extended Table** B5 Supplement A, Fig. 3c).

The best MLP to predict medPOD relied on unimodal EHRs such as mean blood pressure (abs MW = 0.186) or administered fluid volume (abs MW = 0.183). The MLP focused on intraoperative norepinephrine infusion (abs MW = 0.172), the tidal volume (abs MW = 0.166), and heart frequency (abs MW = 0.142). Additionally, preoperative CRP levels (abs MW = 0.158), physical status (ASA abs MW = 0.120), hematocrit (abs MW = 0.135), or erythrocyte count (abs MW = 0.133) had predictive values (see **Extended Table A4** in Supplement B). Highest MWs regarding MRIs for medPOD, provided by the unimodal MLP, were found for the thalamus volume (abs 0.27) (see Extended Table B5 in Supplement A). Additional analyses of the relationship between key covariates and model raw output probabilities for each surgery did not suggest model biases towards gender or age (see **Extended Figure B6**,** Extended Results B3** in Supplement A).

## Discussion

We are the first to demonstrate that neuroanatomical atrophy measures contribute to successful multimodal POD predictions in a general surgical patient population. In contrast to previous studies, our study leveraged data from clinical routinely-collected MRIs which are heterogenous and noisy, but proved to hold potential for clinical decision making^10,35,36^. Best unimodal prediction using MRI morphometry measures achieved 72% AUROC expressing the highest predictive value for subcortical volumetric measures such as the thalamus. Through the iterative application of diverse data fusion strategies and multiple ML models, we achieved high performances up to 86% AUROC for multimodal models, where frontal and temporal cortical atrophy were highly predictive.

Our findings provide clinical utility by enabling preoperative risk stratification that leverages routinely acquired MRI alongside EHR data. In less critically ill patients, cortical atrophy (frontal/temporal) flags intrinsic brain vulnerability to delirium, supporting early initiation of clinical delirium-prevention bundles (e.g., reorientation, sleep protection, mobilization). In higher-acuity settings, reflected by medPOD, the multimodal model emphasizes systemic factors (e.g., anemia, hydration, infection proxies), guiding optimization before surgery (e.g., hemoglobin targets, volume status, infection control) and postoperative monitoring. Multimodal approaches, such as future MRI-informed perioperative decision-making tools, have the potential to improve prevention, triage, and targeted management of POD while complementing clinician judgment and existing care pathways.

We assessed the predictive properties of neuroanatomical and clinical markers in two cohorts based on different POD endpoints. In addition to validated clinical POD-assessment methods with Nu-DESC and CAM-ICU scores (scoPOD), we defined POD in a subgroup of ICU patients according to agitation and pharmacotherapy (medPOD). Importantly, both endpoints highly correlated with the documented ICD diagnosis of delirium, but reflected distinct subgroups with key differences in clinical and surgical characteristics. While the score-based cohort scoPOD covered a wider range of surgical interventions, the smaller medPOD cohort had higher degrees of critical illness and systemic inflammation. Findings of scoPOD associated cortical thickness parameters in the temporal and frontal lobe, involved in memory, attention, and higher-order executive functions, align with literature linking temporal cortex atrophy to delirium^37^. These cortical features outperformed EHR predictors, suggesting that brain-specific vulnerability, reflected by these morphometric measures, is primary driving POD in less critically ill populations. When illness is more critical and patients in intensive care receive antipsychotic pharmacotherapy, MRI measures emphasize the importance of fronto-striato-thalamic circuits in disorders of consciousness and the emergence of psychotic symptoms^38^. However, such subcortical brain vulnerability measures are outweighed by EHR features when POD is predicted in more complex critical illness. Here, preoperative anemia, hydration and infection proxies, significantly associated with POD in univariate statistics, also showed high predictive value.

To keep the clinical scope wide, the presented models are more generalizable compared to previous prediction studies based on clinical imaging of the hippocampus in cardiovascular surgeries^39^. While previous work trained ML on EHRs only^40,41^, we could show that the combination of data modalities improved prediction performances, especially for a less critically ill population.

For explainable artificial intelligence (XAI) purposes, we preferred directly reading model weights over methods like Shapely or LIME due to their susceptibility to unfavorable effects such as suppressor variables^42^. Since model weights are technical properties, we provide comprehensive univariate analyses to enhance clinical insights. To correct for strong confounders, such as age in neuroanatomical measures, we applied linear mixed-effects models focusing on such covariates^5^. However, due to the highly inter-correlated nature of our data, latent noise may not be fully excluded.

Although there are numerous indications for preoperative cranial MRI-scans, most patients who received cranial MRI comprised neurosurgical cases in our sample. In line with previous findings^43,44^, neurosurgical procedures were not predictive for POD in the larger scoPOD sample, nor did we find notably higher POD prevalence rates in our cohorts. However, in the smaller cohort of intensive care patients, the presence of a neurosurgical procedure was significantly associated with POD. While this needs to be replicated, we speculate that through disease severity, patients who undergo major neurosurgical procedures and who require intensive care might be especially vulnerable to POD. Critically, we did not find model biases regarding age, urgency class, sex, or performed neurosurgery suggesting a broad applicability of our results.

Since brain morphometry measures were extracted from heterogenous clinical MRI assessments, we performed quality control to identify anatomical aberrations and segmentation inaccuracies. Automated morphometry tools, such as Freesurfer, are optimized for non-contrast enhanced (CE) images. However, excellent reliability and agreement is reported for T1wCE segmentation^26^.

The presented work has several limitations. Only healthy hemispheres without visually detectable structural lesions interfering with segmentation accuracy were included in our analyses, potentially excluding valuable data. Future work may incorporate more automated and data-driven segmentation approaches to optimize the utilization of clinical scans with potential lesions enhancing prediction robustness. The sample size, particularly for medPOD, restricts generalizability and findings should be validated in larger external cohorts. Hypoactive delirium which is often undiagnosed was not separately addressed in our prediction targets but we aim to formulate a multi-class prediction problem in the future. As with most real-world clinical data, the EHRs used in this study were not primarily collected for secondary research purposes. Consequently, institution-specific documentation practices and local clinical guidelines may have introduced biases and data quality limitations. We inherently handled class-imbalance by robust MWU test statistics and a weighted BCE loss. Oversampling techniques may have resulted in different findings. Reporting AUPRC metrics, which are sensitive to class-imbalances, enabled a more elaborate assessment of model performance compared to exclusively citing AUROC scores^45^. In contrast to randomized controlled trials, causality cannot be assumed for identified relationships while future work aims to include causal inference methods like propensity scores to increase reliability.

In conclusion, this study highlights the advantages of multimodal fusion models that integrate routine MRI and EHR data, harnessing the potential of modern machine learning for outcome prediction. Additionally, this study demonstrates the added value of MRI data in supporting clinical decision-making and improving the management of postoperative delirium in the future.

## Supplementary Information

Below is the link to the electronic supplementary material.

Supplementary Material 1

Supplementary Material 2

Supplementary Material 3

## Data Availability

Python code can be accessed via [https://github.com/ngiesa/fusion_pod](https:/github.com/ngiesa/fusion_pod). We provide comprehensive summary statistics of patient data in the Supplement A. The concrete datasets analyzed during the current study are not publicly available due German data privacy regulations, but are available from the corresponding author on reasonable request. We report results according to the “Transparent reporting of a multivariable prediction model for individual prognosis or diagnosis” (TRIPOD) guidelines (see **Extended Table** A5 in Supplement B).

## Abbreviations

MRI: Magnet resonance imaging
AUROC: Are under receiver operating characteristics
AUPRC: Area under precision recall curve
BCE: Binary cross-entropy
CIS: Clinical information system
CAM: Confusion assessment method
CE: Contrast-enhanced
CV: Cross validation
DWH: Data warehouse
HER: Electronic health record
XAI: Explainable artificial intelligence
FDR: False discovery rate
GBT: Gradient boosted trees
ICU: Intensive care unit
ICD: International classification of diseases
LR: Logistic regression
ML: Machine learning
MWU: Mann Whitney U
MEM: Mixed-effects model
MLP: Multi-layer perceptron
OR: Odds ratio
PACS: Picture archiving and communication system
POD: Postoperative delirium
SOFA: Sequential organ failure

## Declarations

This study was approved by the Ethics Committee of the Charité Universitätsmedizin – Berlin (EA2/024/18) and followed the Declaration of Helsinki. Patient consent for general research purpose was covered by the patient treatment contract. Specific analysis of patient data approved the IRB EA2/024/18.
