# Supplementary material for: Fusion of clinical magnet resonance images and electronic health records promotes multimodal predictions of postoperative delirium
Source: Sci Rep. 2025 Dec 26;15:44654. doi: 10.1038/s41598-025-31693-9 (PMC12749217; doi:10.1038/s41598-025-31693-9)
Supplement: Supplementary file 1 — Supplementary Material 1 [file 41598_2025_31693_MOESM1_ESM.docx]

**Supplement A for the manuscript “Fusion of clinical magnet resonance images and electronic health records promotes multimodal predictions of postoperative delirium” by Niklas Giesa et al.**


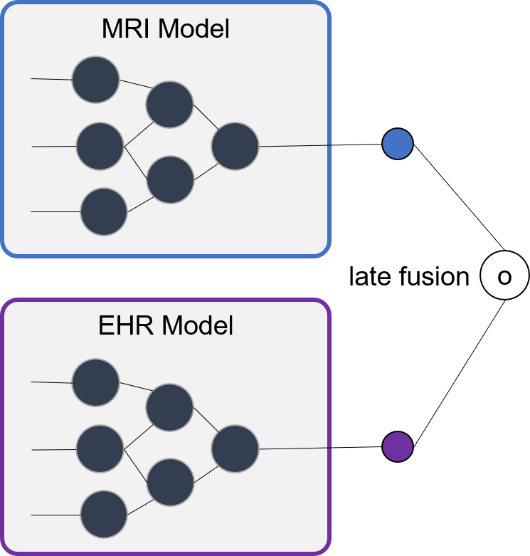


**Extended Figure B1:** Visualization of late fusion strategy where two models are trained for with MRI and EHR inputs separately, the combined prediction results “o” is either averaged for LR and BT models, or combined through a linear layer for backpropagation in MLP networks (joint fusion).

| **Endpoint** | **Model** | **Fusion** | **Hyperparameters** |
| --- | --- | --- | --- |
| scoPOD | LR | MRI only | L1 penalty of 1 |
|  | BT |  | 10 estimators, max depth 2 |
|  | MLP |  | Nodes per layer [2,2,2] |
|  | LR | EHR only | L1 penalty of 1E-10 |
|  | BT |  | 100 estimators, max depth 1 |
|  | MLP |  | Nodes per layer [4,4,4,4] |
|  | LR | early fusion | L1 penalty of 1E-10 |
|  | BT |  | 10 estimators, max depth 2 |
|  | MLP |  | Nodes per layer [4,4,4,4] |
|  | LR | late fusion | L1 penalty of 1E-10 |
|  | BT |  | 10 estimators, max depth 2 |
|  | MLP |  | Nodes per layer [8,8,8,8] |
| medPOD | LR | MRI only | L1 penalty of 1E-10 |
|  | BT |  | 100 estimators, max depth 1 |
|  | MLP |  | Nodes per layer [4,4,4,4] |
|  | LR | EHR only | L1 penalty of 1 |
|  | BT |  | 10 estimators, max depth 2 |
|  | MLP |  | Nodes per layer [4,4,4,4] |
|  | LR | early fusion | L1 penalty of 1E-10 |
|  | BT |  | 100 estimators, max depth 1 |
|  | MLP |  | Nodes per layer [2,2,2] |
|  | LR | late fusion | L1 penalty of 1E-10 |
|  | BT |  | 10 estimators, max depth 2 |
|  | MLP |  | Nodes per layer [2,2,2] |

**Extended Table B2:** Model hyperparameters trained through a Grid-Search approach. Models are trained with log-loss (LR, BT) or cross-entropy (MLP), learning rate was at 1e-4 with 20 epochs to train for MLPs. Hyperparameters resulting into the smallest testing loss within nested cross-validation were selected.

|  | **Endpoint Definition 1**  **scoPOD (n=645)** | | **Endpoint Definition 2**  **medPOD (n=224)** | |
| --- | --- | --- | --- | --- |
|  | **Descriptive Statistics [all]** | **Adjusted**  **P-Value [POD]** | **Descriptive Statistics [all]** | **Adjusted**  **P-Value [POD]** |
| **OPS Procedure** |  |  |  |  |
| Nervous System | 0.68 (438/207) | 3.66E-02 | 0.68 (153/71) | **1.39E-04*** |
| Dermatology | 0.27 (171/474) | 1.89E-03 | 0.25 (56/168) | 1.29E-01 |
| Orthopedic | 0.23 (147/498) | 4.68E-01 | 0.23 (51/173) | 1.53E-03 |
| Gastrointestinal | 0.11 (67/568) | 5.46E-03 | 0.15 (34/190) | 2.18E-03 |
| Head | 0.14 (94/551) | 7.60E-01 | 0.16 (35/189) | **1.44E-09*** |
| Vascular | 0.11 (69/576) | 8.10E-01 | 0.09 (21/203) | **4.14E-09*** |
| Endocrine | 0.05 (33/612) | 7.39E-01 | 0.08 (17/207) | 7.62E-01 |
| Thorax | 0.04 (30/615) | 1.14E-02 | 0.07 (16/208) | **2.69E-05*** |
| Urology | 0.04 (29/616) | 9.75E-04 | 0.06 (15/209) | 1.30E-02 |
| Gynaecology | 0.03 (19/626) | 5.93E-03 | 0.05 (11/213) | 3.83E-01 |

**Extended Table B3**: OPS (Operationen und Prozeduren Schlüssel) codes as documenting the surgical procedures that were performed. OPS are procedure codes applied in the German health system. Significant results according to a FDR corrected alpha level are marked in boldness and with an asterisks.

| **endpoint** | **fusion** | **model** | **CI lower AUROC** | **Mean AUROC** | **CI upper AUROC** | **CI lower AUPRC** | **Mean AUPRC** | **CI upper AUPRC** |
| --- | --- | --- | --- | --- | --- | --- | --- | --- |
| scoPOD | EHR only | LR | 0.769 | 0.781 | 0.794 | 0.524 | 0.547 | 0.57 |
|  |  | BT | 0.825 | 0.835 | 0.845 | 0.575 | 0.597 | 0.618 |
|  |  | MLP | 0.851 | 0.861 | 0.871 | 0.644 | 0.665 | 0.687 |
|  | early fusion | LR | 0.764 | 0.776 | 0.789 | 0.492 | 0.516 | 0.541 |
|  |  | BT | 0.828 | 0.838 | 0.849 | 0.595 | 0.619 | 0.643 |
|  |  | MLP | 0.847 | 0.857 | 0.867 | 0.636 | 0.657 | 0.679 |
|  | late fusion | LR | 0.775 | 0.788 | 0.8 | 0.517 | 0.541 | 0.565 |
|  |  | BT | 0.831 | 0.841 | 0.851 | 0.574 | 0.597 | 0.62 |
|  |  | MLP | 0.847 | 0.857 | 0.867 | 0.619 | 0.643 | 0.668 |
|  | MRI only | LR | 0.681 | 0.694 | 0.706 | 0.376 | 0.394 | 0.412 |
|  |  | BT | 0.682 | 0.696 | 0.711 | 0.401 | 0.423 | 0.445 |
|  |  | MLP | 0.703 | 0.717 | 0.731 | 0.427 | 0.449 | 0.47 |
| medPOD | EHR only | LR | 0.667 | 0.677 | 0.687 | 0.388 | 0.404 | 0.419 |
|  |  | BT | 0.677 | 0.686 | 0.696 | 0.383 | 0.399 | 0.415 |
|  |  | MLP | 0.703 | 0.713 | 0.722 | 0.423 | 0.438 | 0.453 |
|  | early fusion | LR | 0.684 | 0.694 | 0.704 | 0.372 | 0.387 | 0.401 |
|  |  | BT | 0.697 | 0.707 | 0.717 | 0.402 | 0.418 | 0.433 |
|  |  | MLP | 0.722 | 0.731 | 0.74 | 0.409 | 0.424 | 0.439 |
|  | late fusion | LR | 0.695 | 0.705 | 0.715 | 0.389 | 0.404 | 0.42 |
|  |  | BT | 0.712 | 0.722 | 0.732 | 0.436 | 0.450 | 0.468 |
|  |  | MLP | 0.726 | 0.735 | 0.744 | 0.441 | 0.458 | 0.477 |
|  | MRI only | LR | 0.632 | 0.643 | 0.653 | 0.251 | 0.261 | 0.271 |
|  |  | BT | 0.625 | 0.635 | 0.644 | 0.304 | 0.317 | 0.331 |
|  |  | MLP | 0.666 | 0.675 | 0.685 | 0.325 | 0.339 | 0.34 |

**Extended Table B4**: Final testing performance metrics in terms of AUROC and AURPC scores. We report mean scores, and the upper, lower bounds of the 95^th^ confidence interval (CI) across 1000x bootstrapped testing sets performed on hold-out cross-validation folds.

| **endpoint** | **MRI feature** | **model weight** |
| --- | --- | --- |
| scoPOD | Temporal pole thickness | 0.565 |
|  | Superior frontal thickness | 0.523 |
|  | Insula thickness | 0.504 |
|  | Middle temporal thickness | 0.409 |
|  | Superior temporal thickness | -0.052 |
| medPOD | Thalamus | -0.27 |
|  | Fusifom Area | 0.206 |
|  | Inferior temporal area | 0.195 |
|  | Ventral diencephalon | -0.186 |
|  | Posterior cingulate volume | 0.174 |
|  | Brainstem | -0.17 |
|  | Insula area | 0.17 |
|  | Entorhinal area | -0.153 |
|  | Subcortical gray matter volume | 0.134 |
|  | Caudal anterior cingulate thickness | 0.125 |

**Extended Table B5:** Model weights from MLP trained with MRI features only and applied l_1_ regularization term. For scoPOD all selected model weights are shown, for medPOD the top 10 features are included.


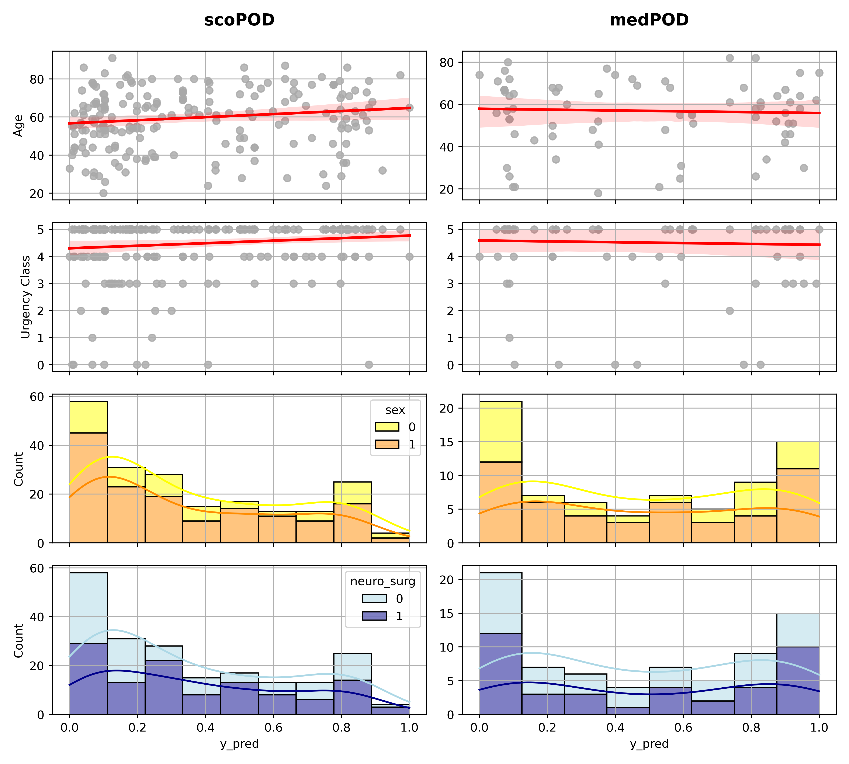


**Figure B6: Model outputs in relation to parameters potentially inducing bias**. The models output probability (y_pred, on x-axis) is displayed alongside 1) numerical variables (Age, Urgency Class) where each point corresponds to one surgery / prediction, or 2) distribution of two classes (sex, neuro_surg) drawn as corresponding histograms. Red line represents a linear regression fit in 1), colored lines for 2) approximate the kernel density

**Extended Results B1**

The medPOD cohort covered slightly younger patients than scoPOD (Δ of mean age = 1.92 years), both included more male patients than females (78 and 69%). The presence of ICD encoded renal disease (ln(OR) = 1.13, p-value = 3.19E-07 for scoPOD, ln(OR) = 1.47, p-value = 3.24E-06 for medPOD) and SIRS (ln(OR) = 0.92, p-value = 7.96E-05 for scoPOD, ln(OR) = 2.36, p-value = 2.99E-20 for medPOD) were significantly more often found in patients with POD for both endpoints. The presence of sepsis was significant for medPOD with ln(OR) = 2.29 and p-value = 5.94E-13.

**Extended Results B2**

Figure B6 depicts our investigated model covariates in relation to raw model output probabilities. Correlations of numerical variables, like age, with POD are shown as scatter charts, dichotomous covariates are shown as histograms. Again, we applied the MWU or Spearman correlations for investigating significance. We did not find evidence of strong correlation between age (scoPod p-value = 7.676E-2, medPOD p-value = 9.880E-1), urgency class (scoPod p-value = 1.217-2, medPOD p-value = 7.776E-1), sex (scoPod p-value = 7.511E-1, medPOD p-value = 9.589E-1), or performed neurosurgery (scoPod p-value = 2.753-1, medPOD p-value = 6.856E-1).

Additionally, age MW for both cohorts were relatively low ranked with 0.033 for scoPOD and under 0.043 for medPOD, so model features with combined model variants preferred other trajectories than age. Hence, we concluded that our models are not substantially biased towards these variables of interest.
